# Person-Environment-Occupation Model of Mealtime Performance in Older People With Dementia: Theory Analysis

**DOI:** 10.1093/geroni/igaf122.3187

**Published:** 2025-12-31

**Authors:** Zih-Ling Wang, Basia Belza

**Affiliations:** University of Washington School of Nursing, Seattle, Washington, United States; University of Washington, Seattle, Washington, United States

## Abstract

Mealtime challenges are prevalent among people living with dementia (PLWD) and significantly affect their quality of life and nutritional status. Mealtime performance differs from concepts like “eating behaviors” or “feeding difficulties” by focusing on the holistic functional capacity rather than isolated challenges. This paper presents a theory analysis of the Person-Environment-Occupation (PEO) Model as applied to mealtime performance in older people with dementia, using Walker and Avant’s systematic approach. The PEO Model provides a holistic framework for understanding the dynamic interactions between individuals, their environments, and their occupations. The analysis examines the model’s origins, meaning, logical adequacy, usefulness, generalizability, parsimony, and testability in the context of mealtime performance for PLWD. Findings indicate that the PEO Model effectively conceptualizes how personal factors, environmental factors, and occupational factors interact to influence mealtime performance. The transactions between person-environment, environment-occupation, and person-occupation provide valuable insights into barriers and facilitators of successful mealtime engagement. While the PEO model has been extensively used as a framework in occupational therapy practice, it has yet to be fully applied within nursing science, particularly in dementia care. This model has potential applications in long-term care settings, where environmental modifications and personalized approaches can enhance mealtime participation. This analysis contributes to nursing science by providing a theoretical foundation for developing person-centered interventions for PLWD across different care settings.

